# HIV-1 molecular transmission network among sexually transmitted populations in Liaoning Province, China

**DOI:** 10.1097/MD.0000000000026640

**Published:** 2021-07-16

**Authors:** Ning Ma, Xing-hua Chen, Yan Zhao, Xu Kang, Shan Pan, Wen-qing Yao

**Affiliations:** a Liaoning Provincial Center for Disease Control and Prevention, Shenyang, Liaoning, China; b The First Hospital of China Medical University, Shenyang, Liaoning, China.

**Keywords:** gene subtype, human immunodeficiency virus-1, molecular transmission network, sexually transmitted

## Abstract

**Introduction:**

In recent years, with the development of molecular epidemiology, molecular transmission networks based on evolutionary theory and sequence analysis have been widely used in research on human immunodeficiency virus (HIV)-1 transmission dynamics and precise intervention for high-risk populations. The HIV-1 molecular transmission network is a new method to study the population's access to the network, the characteristics of clustering, and the characteristics of interconnection in the network. Here, we analyzed the characteristics of the HIV-1 molecular transmission network of sexually transmitted people in Liaoning Province.

**Methods:**

A study of HIV-infected persons who were sexually transmitted in Liaoning Province from 2003 to 2019. HIV-1 RNA was extracted, amplified and sequenced, and a phylogenetic tree was constructed to determine the subtype using the well matched *pol* gene region sequence. The gene distance between sequences was calculated, the threshold was determined, and the molecular transmission network was constructed.

**Results:**

109 samples of *pol* gene region were obtained. The main subtype of HIV-1 was CRF01_AE, followed by B, CRF07_BC, etc. 12.8% of them were resistant to HIV. At the threshold of 0.55 gene distance, 60.6% of them entered the HIV-1 molecular transmission network. Workers, sample source voluntary counseling and testing, other testing, subtype B and drug resistance are the factors influencing the access to HIV-1 molecular transmission network. The subtype of CRF01_AE formed 6 clusters in the molecular transmission network. In the network, the difference of connection degree between different subtypes was statistically significant.

**Discussion:**

The three subtypes CRF01_AE, CRF07_BC and B that enter the molecular transmission network do not have interconnections, and they form clusters with each other. It shows that the risk of transmission among the three subtypes is less than the risk of transmission within each subtype. The factors affecting HIV-1 entry into the molecular transmission network were occupation, sample source, genotype and drug resistance. The L33F mutation at the HIV-1 resistance mutation site constitutes the interconnection in the largest transmission cluster in the network. The epidemiological characteristics of HIV-infected persons in each molecular transmission cluster show that 97% of the study subjects come from the same area and have a certain spatial aggregation.

**Conclusion:**

Constructing a molecular transmission network and conducting long-term monitoring, while taking targeted measures to block the spread of HIV can achieve precise prevention and control.

## 1 Introduction

The Human Immunodeficiency Virus/Acquired Immune Deficiency Syndrome (HIV/ acquired immune deficiency syndrome) epidemic has become one of the most critical issues that seriously affect public health. Since the start of the epidemic, around 75 million people have been infected with HIV, and in 2018, 37.9 million people were living with HIV worldwide.^[1]^ By the end of October 2019, approximately 958 thousand people lived with HIV in China.^[2]^ In recent years, with the development of molecular epidemiology, molecular transmission networks based on evolutionary theory and sequence analysis have been widely used in the study of HIV-1 transmission kinetics and precision intervention in high-risk populations,^[3–5]^ especially HIV -1 Long-term monitoring of drug resistance and real-time prevention interventions.^[6,7]^ The HIV-1 molecular transmission network refers to a group of sequences that are not randomly gathered and have a certain epidemiological correlation. It constructs a transmission network through the genetic information of people infected with HIV through similar viruses with similarities and connections, and restores the macro social network of infected people as much as possible, which aims to focus on analyzing the characteristics of infected people, and preventing and controlling the active and critical groups in the network.^[8,9]^ HIV is transmitted through networks formed by closely connected individuals who engage in injecting or sexual behaviours.^[10,11]^ However, the research on HIV-1 molecular cluster-based transmission network in China is still in its infancy, and only some regions have relevant reports.^[12–14]^ Based on traditional epidemiological methods such as questionnaires, peer tracking and disease surveillance, molecular transmission network analysis provides a new method for the analysis of HIV-1 network characteristics at the population level.^[15]^ HIV-1 molecular transmission network can reflect the social transmission network of HIV-1 to a certain extent according to the network access, clustering characteristics and interconnection characteristics of the study population. Promotion and analysis of molecular cluster-based communication network analysis techniques and strategies are mainly to further contribute to traditional social communication network analysis.^[16]^ In this study, we selected the sexually transmitted HIV-infected persons in Liaoning Province, using molecular epidemiological analysis, supplemented by field epidemiological data, to reveal the distribution of HIV-1 subtypes and molecular transmission network in Liaoning Province, China.

## 2 Methods

### 2.1 Study subjects

In our study, patients who were diagnosed with HIV between 2003 and 2019 were selected from the “ acquired immune deficiency syndrome integrated prevention and treatment information system” of the Chinese Center for Disease Control and Prevention. The present address of these HIV-infected persons is Liaoning Province. 117 of them had not received antiviral treatment. From these individuals, 109 sexually transmitted HIV-infected individuals were screened for the study. Demographic and behavioral data were collected for these 109 individuals, while their blood samples were collected. All of these subjects signed informed consent.

### 2.2 Methods

#### 2.2.1 Nucleic acid extraction, amplification and sequencing

CD4^+^ T lymphocyte cell counts were completed within 24 hours from the collected whole blood samples. The plasma was then separated at 1500 rpm for 15 minutes using a Beckman-Kurt centrifuge. The plasma was packed and frozen at −70°C. The viral load in plasma was determined using Roche TaqMan 48 and matched HIV viral load detection kit. HIV-1 RNA was extracted from plasma using QIAampRNA MiniKit reagent from QIAGEN. Nested reverse transcription fluorescence quantitative polymerase chain reaction amplification was performed on 1.2 KB long fragment of *pol* region gene (1–99 codons in protease region and 1–250 codons in reverse transcriptase region) by In-house method. The amplified products were identified by agarose gel electrophoresis imaging. The amplified positive products were sent to Beijing Bomeid Gene Technology Co. Ltd. for purification and gene sequencing. Sanger sequencing method was used. Sequencing primers refer to “HIV resistance monitoring strategy and detection technology.”^[17]^

#### 2.2.2 Sequence analysis

The measured sequence results were spliced and cleaned with the software Chromas1.62. Sequences were corrected with BioEdit 7.0. And compared with the international reference sequence (Los Alamos National Laboratory HIV Sequence Database). The aligned sequences were used to construct phylogenetic trees using the neighbor-joining method in MEGA7.0. The corresponding gene subtypes were determined by clustering with international reference strains. Using the online analysis tool COMET (https://comet.1ih.lu/index.php) Review. The aligned sequences were imported into the HIV resistance database of Stanford University (USA). http://hivdb.Stanford.edu) Online analysis of resistance mutation sites.

#### 2.2.3 Molecular propagation network analysis

The aligned sequences were imported into MEGA7.0 software. Gene distances between all pairs were calculated using the Tamura-Nei93 model. The Bootstrap value of branch nodes of phylogenetic tree species is ≥90%, the number of samples within the cluster is ≥2, and the average gene distance within the cluster is less than or equal to the set threshold, which is defined as a molecular propagation cluster.^[18]^ By observing the total number of propagation clusters in the propagation network under a series of thresholds, it is found that the total number of propagation clusters in the network reaches a peak (9) when the threshold (i.e., the gene threshold when the propagation cluster is the largest and the sample size included is the largest) is 0.55. Therefore, a molecular propagation network was constructed using Cytoscape 3.7.2 software with 0.55 as the threshold to identify potential propagation partners. The degree is the degree of association. Represents the number of edge connections between a node and other nodes in a molecular propagation network. Access rate is the percentage of the total number of people entering the molecular transmission network.

### 2.3 Statistical analysis

Statistical analysis was performed using SPSS19.0 and Excel 2010. Count data are expressed in frequency. *χ*^2^ test was used to analyze the correlation between two categorical variables. Univariate and multivariate logistic regression models were used to analyze the influencing factors of the access rate. *P* *<* .05 indicated that the difference was statistically significant.

## 3 Results

### 3.1 Basic information of the study subjects

A total of 109 samples that passed through sexual transmission route and successfully obtained *pol* gene region sequence were screened out. Among them, 102 (93.6%) were male, the median age was 39 years old (interquartile range, [IQR], 29–52), 96 (88.1%) were Han nationality, 42 (38.5%) were junior high school and below culture, 53 (48.6%) were unmarried, 92 (84.4%) were living locally, 30 (27.5%) were housekeeping, housework and unemployment, 83 (76.1%) were homosexual transmission, 181 CD4^+^ cells/ul (IQR, 62–260), and the HIV viral load was 49700 Copies/ml (IQR, 13950–147000). See Table 1 for details.

**Table 1:** Demographic characteristics of the study objects.

Project	Median (IQR)	Frequency (%)
Gender		
Male		102 (93.6)
Female		7 (6.4)
Age	39 (28-52)	
Ethnicity		
Han		96 (88.1)
Others		13 (11.9)
Educational level		
Junior high school and below		42 (38.5)
High school or technical secondary school		28 (25.7)
College and above		39 (35.8)
Marital status		
Unmarried		53 (48.6)
Married with spouse		27 (24.8)
Divorced or widowed		29 (26.6)
Place of residence		
Local		92 (84.4)
Non local		17 (15.6)
Occupation		
Housekeeping, housework and job hunting		30 (27.5)
Worker		10 (9.2)
Business waiter		7 (6.4)
Cadre staff		10 (9.2)
Retired		10 (9.2)
Farmer		9 (8.3)
Others and unknown		33 (30.3)
Route of infection		
Male same-sex transmission		83 (76.1)
Heterosexual transmission		26 (23.9)
Sample source		
Testing Consulting		32 (29.4)
STD clinic		22 (20.2)
Other visitor tests		29 (26.6)
Other sources		26 (23.9)
CD4 cells (cells /ul)	181 (62-260)	
HIV viral load (copies /ml)	49700 (13950-147000)	

### 3.2 HIV-1 subtype analysis

**Figure 1:**
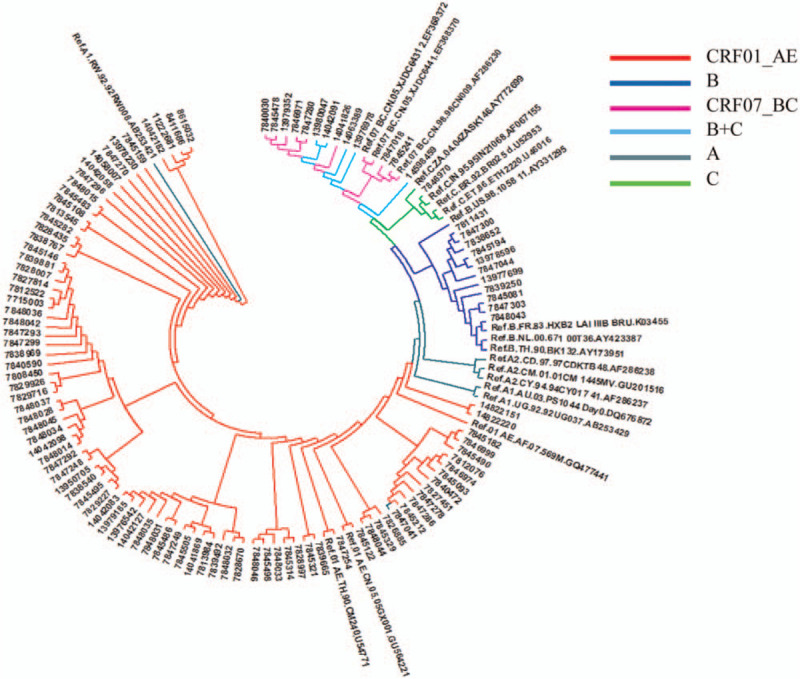
Phylogenetic tree of HIV-1 subtype adjacency method. HIV = human immunodeficiency virus.

The HIV-1 subtypes of the subjects were mainly CRF01_AE subtype, 83 cases (76.1%), followed by B subtype, 11 cases (10.1%), CRF07_BC subtype, 8 (7.3%), B+C subtype, 5 (4.6%), A subtype and C subtype, 1 (0.9%) respectively, see Figure 1. A distinct aggregation phenomenon was formed on the phylogenetic tree. CRF01_AE subtype, average genetic distance was 0.332 ± 0.057, average genetic distance between B subtypes was 0.032 ± 0.004, CRF07_BC subtypes, average genetic distance was 0.012 ± 0.002, and between B+C subtypes was 0.026 ± 0.004.

Fourteen (12.8%) of the subjects developed HIV resistance. Among them, 9 cases were protease inhibitor-related resistance, and the main resistance sites were L33F, V82A, Q58E, M46L, M46I; There were 4 cases of nucleoside reverse transcriptase inhibitor resistance, the main mutation sites were V75VI, K219Q, T215A, K65R; There were 5 cases of non-nucleoside reverse transcriptase inhibitor resistance, the main mutation sites were V179E, A98G, V106I, Y181C, G190S, V179D. Drug resistance and mutation sites in 14 patients are shown in Table 2.

**Table 2:** Drug resistance and mutation sites in 14 patients.

		Potential Low-Level Resistance		Low-Level Resistance		Intermediate Resistance		High-Level Resistance	
Drug Classification		Number of cases	Mutations	Number of cases	Mutations	Number of cases	Mutations	Number of cases	Mutations
Protease Inhibitors	atazanavir/r (ATV/r)	2	M46L|M46I	1	V82A				
	fosamprenavir/r (FPV/r)	5	L33F|V82A|M46L|M46I						
	indinavir/r (IDV/r)	2	M46L|M46I			1	V82A		
	lopinavir/r (LPV/r)	2	M46L|M46I	1	V82A				
	saquinavir/r (SQV/r)	2	V82A|M46I						
	nelfinavir (NFV)			2	V82A|M46L	2	V82A|M46I		
	tipranavir/r (TPV/r)	2	L33F|Q58E						
Nucleoside Reverse Transcriptase Inhibitors	lamivudine (3TC)					1	K65R		
	abacavir (ABC)					1	K65R		
	zidovudine (AZT)	1	K219Q	1	T215A				
	stavudine (D4T)	2	V75VI|K219Q	1	T215A			1	K65R
	didanosine (DDI)	1	V75VI					1	K65R
	emtricitabine (FTC)					1	K65R		
	tenofovir (TDF)							1	K65R
Non-nucleoside Reverse Transcriptase Inhibitors	efavirenz (EFV)	2	V179D	1	A98G			1	Y181C+G190S
	etravirine (ETR)	3	A98G|V106I|V179D			1	Y181C+G190S		
	nevirapine (NVP)	3	V179E|V106I|V179D			1	A98G	1	Y181C+G190S
	rilpivirine (RPV)	2	V106I|V179D	1	A98G			1	Y181C+G190S

### 3.3 Molecular propagation network analysis

3.3.1 HIV-1 Molecular Transmission Rate and Its Influencing Factors

**Figure 2:**
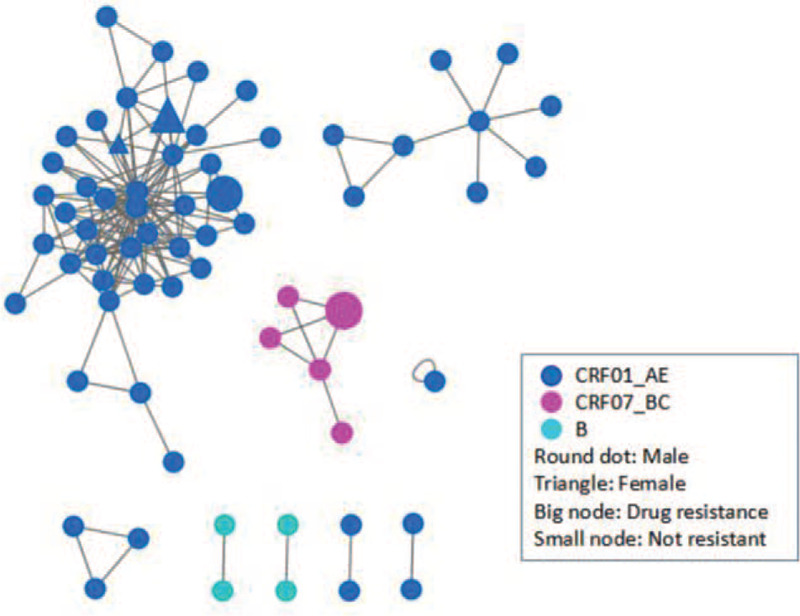
HIV-1 molecular transmission network of sexually transmitted populations in Liaoning Province, China. HIV = human immunodeficiency virus.

Under the 0.55 gene distance threshold, a total of 66 series entered the HIV-1 molecular propagation network, with a total access rate of 60.6% (Fig. 2).

Whether to enter the HIV-1 molecular transmission network is taken as the dependent variable. Univariate and multivariate logistic regression analysis was performed with sex, age, ethnicity, educational level, marital status, residence, occupation, route of infection, source of samples, genotype and drug resistance as independent variables (Table 3). The results showed that worker, sample source testing consultation and other visiter testing, subtype B and drug resistance were the influencing factors for entering the HIV-1 molecular transmission network.

**Table 3:** Analysis of the influencing factors of HIV-1 entering the molecular transmission network of sexually transmitted people in Liaoning Province.

			One-factor logistic analysis		Multifactor Logistic Analysis	
Project	Total	Internet access rate (%)	*OR (95% CI)*	*P* value	*aOR (95% CI)*	*P* value
Gender						
Male	102	64 (62.7)	0.238 (0.044-1.285)	.095		
Female	7	2 (28.6)	1			
Age						
≤30	36	22 (61.1)	1			
31-50	44	25 (56.8)	1.194 (0.497–2.929)	.698		
> 50	29	19 (65.5)	0.287 (0.299–2.288)	.715		
Ethnicity						
Han	96	60 (62.5)	1			
Others	13	6 (46.2)	1.944 (0.606–6.241)	.264		
Educational level						
Junior high school and below	42	26 (61.9)	1.099 (0.445–2.712)	.838		
High school or technical secondary school	28	15 (53.6)	1.548 (0.575–4.164)	.387		
College and above	39	25 (64.1)	1			
Marital status						
Unmarried	53	30 (56.6)	0.944 (0.379–2.347)	.901		
Married with spouse	30	20 (66.7)	0.431 (0.139–1.333)	.144		
Divorced or widowed	29	16 (55.2)	1			
Place of residence						
Local	92	55 (59.8)	1.233 (0.419–3.626)	.703		
Nonlocal	17	11 (64.7)	1			
Occupation						
Housekeeping, housework and job hunting	30	14 (46.7)	3.571 (1.223–10.429)	.020	3.018 (0.810–11.239)	.100
Worker	10	4 (40.4)	4.687 (1.051–20.899)	.043	7.154 (1.057–48.417)	**.044**
Business waiter	7	2 (28.6)	7.812 (1.262–48.356)	.027	8.778 (0.898–85.767)	.062
Cadre staff	10	7 (70.0)	1.339 (0.279–6.434)	.715	1.156 (0.185–7.214)	.877
Retired	10	8 (80.0)	0.781 (0.137–4.460)	.781	0.617 (0.068–5.594)	.668
Farmer	9	6 (66.7)	1.562 (0.316–7.726)	.584	3.228 (0.430–24.252)	.255
Other and unknown	33	25 (75.8)	1		1	
Route of infection						
Same-sex transmission	83	51 (61.4)	0.856 (0.350–2.094)	.733		
Heterosexual transmission	26	15 (57.7)	1			
Sample source						
Testing Consulting	32	14 (43.8)	7.071 (1.978–25.278)	.003	6.984 (1.476–33.043)	**.014**
STD clinic	twenty two	14 (63.6)	3.143 (0.795–12.425)	.103	2.138 (0.415–11.014)	.364
Other visitor tests	29	16 (55.2)	4.469 (1.227–16.275)	.023	6.088 (1.316–28.151)	**.021**
Other sources	26	22 (84.6)	1		1	
HIV genotype						
CRF01_AE	83	57 (68.7)	1		1	
B	11	4 (36.4)	3.837 (1.032–14.263)	.045	7.142 (1.356–37.621)	**.020**
CRF07_BC	8	5 (62.5))	1.315 (0.292–5.923)	.721	2.093 (0.369–11.869)	.404
Others	7	0 (0.0)	—	—	—	—
Whether HIV is resistant						
Resistance	15	3 (20.0)	8.129 (2.137–30.928)	0.002	7.184 (1.286–40.121)	**.025**
Not resistant	94	63 (67.0)	1		1	

### 3.4 Characteristics of HIV-1 molecular transmission network

The CRF01_AE subtype forms six propagation clusters in the molecular propagation network, one of them is the largest propagation cluster in the network, containing 40 nodes. Subtype B forms two propagation clusters, both consisting of two nodes. The CRF07_BC subtype forms a propagation cluster consisting of five nodes. In the network, the median connectivity of CRF01_AE subtypes was 3 degrees (IQR, 2–7). The median connectivity of subtype B is 1 degree (IQR, 1–1), and the median connectivity of CRF07_BC subtype is 2 degrees (IQR, 2–4), the degree of connectivity between different subtypes was statistically significant (*P* < .01). The HIV-1 resistance mutation that enters the network is protease inhibitor-related resistance, which contains 3 nodes, and the mutation sites are L33F and Q58E. In the network, 77.3% had homosexual transmission, 3 degrees of connectivity (IQR, 1–6), 22.7% had heterosexual transmission, and 3 degrees of connectivity (IQR, 2–6). The place of residence is 83.3% in the local area and 16.7% in the field, and the connectivity is 3 degrees (IQR, 1–6).

HIV-infected individuals in the largest molecular transmission cluster are all CRF01_AE subtypes. The genetic distance of the gene was 0.010 ± 0.001. Among them, 27 cases were homosexual transmission and 13 cases were heterosexual transmission. The present address is from 6 urban areas of Shenyang. The second molecular transmission cluster HIV-infected patients were also CRF01_AE subtype. The genetic distance of the gene was 0.012 ± 0.002. They are all homosexual transmission, and their current address is Shenyang City. The characteristics of HIV-infected individuals within other molecular transmission clusters are shown in Table 4.

**Table 4:** Characteristics of HIV infectors/AIDS patients within molecular transmission clusters.

Serial Number	Cases	HIV-1 subtype	Gene distance within transmission cluster	Pathway of infection (n)	Location of address (n)
1	40	CRF01_AE	0.010 ± 0.001	Homosexual transmission (27); Heterosexual transmission (13)	Heping District, Shenyang (12); Shenhe District, Shenyang (3); Dadong District, Shenyang (7); Huanggu District, Shenyang (9); Tiexi District, Shenyang (6); Yuhong District, Shenyang (2); Shuangtaizi District, Panjin (1)
2	9	CRF01_AE	0.012 ± 0.002	Homosexual transmission (9)	Heping District, Shenyang (1); Dadong District, Shenyang (4); Huanggu District, Shenyang (2); Shenbei New District, Shenyang (1); Hunnan District, Shenyang (1)
3	5	CRF07_BC	0.006 ± 0.002	Homosexual transmission (5)	Huanggu District, Shenyang (1); Tiexi District, Shenyang (2); Yuhong District, Shenyang (1); Dawa District, Panjin (1)
4	3	CRF01_AE	0.005 ± 0.002	Homosexual transmission (2); Heterosexual transmission (1)	Shuangtaizi District, Panjin (2); Panshan Town, Panjin (1)
5	2	B	0.004 ± 0.002	Homosexual transmission (2)	Heping District, Shenyang (1); Huanggu District, Shenyang (1)
6	2	B	0.005 ± 0.002	Homosexual transmission (2)	Dadong District, Shenyang (1); Shuangtaizi District, Panjin (1)
7	2	CRF01_AE	0.004 ± 0.002	Homosexual transmission (2)	Xingcheng City, Huludao (2)
8	2	CRF01_AE	0.001 ± 0.001	Homosexual transmission (2)	Heping District, Shenyang (1); Shenbei New District, Shenyang (1)
9	1	CRF01_AE	/	Homosexual transmission (1)	Tiexi District, Shenyang (1)

## 4 Discussion

In this study, the gene sequences and related data of 109 HIV-infected persons sexually transmitted in Liaoning Province from 2003 to 2019 were analyzed. The major genotype was identified as CRF01_AE (76.1%), followed by subtype B (10.1%) and CRF07_BC subtype (7.3%). In this study, a molecular transmission network was constructed according to the gene distance between two sequences calculated by the Tamura-Nei93 model. The Tamura-Nei93 model simulates the conversion and transfer rates of nucleotides and can correct the deviation of HIV substitution and the inconsistency of base composition, taking into account the calculation speed and biological authenticity comprehensively.^[19]^ In this study, we attempted to determine the threshold of gene distance by discriminating the transmission clusters among the sexually transmitted population in Liaoning Province. Under the threshold of 0.55 gene distance, the overall access rate was 60.6%. The higher the access rate, the higher the risk of transmission. The results showed that CRF01_AE access rate is 68.7%, CRF07_BC access rate was 62.5%. And CRF01_AE and CRF07_BC subtypes all formed propagation clusters with more than 2 connectivity degrees. The CRF01_AE subtype forms a large propagation cluster containing 40 nodes in the network. The CRF01_AE subtype mainly exists in homosexual communicators, accounting for 82.4%. This is consistent with Shanghong's report that two CRF01_AE strains were independently introduced into the men who have sex with men (MSM) population in Liaoning Province in the early and middle 1990 s and formed local epidemic clusters consistent.^[20]^ By Shao Yiming team to cover the whole country CRF01_AE Near-full-length sequence analysis was performed on 75 strains from the main endemic areas. Discovery of the CRF01_AE strain in China was introduced via Southeast Asian countries (Thailand) in the 1990 s.^[21]^ The results show that all 15 nodes entering the network in heterosexual propagation are CRF01_AE subtype. Mainly due to the CRF01_AE subtype has been introduced early and has been prevalent for a long time. It has spread from the MSM population to the heterosexual and can form larger transmission clusters. The CRF07_BC subtype forms a propagation cluster consisting of 5 nodes, all of which are homosexual propagation. CRF07_ BC subtype virus was initially formed and mainly prevalent among drug users in China.^[22]^ Previous research by Shang Hong's team suggested that a specific CRF07_BC lineage^[23]^ exists in MSM, and it has a significant foundation in MSM populations across the country. B subtype formed two 1-degree connected clusters composed of MSM populations. Among the earliest reported MSM population in China, B subtype was the main epidemic strain, and some recombinant subtypes containing B subtype components appeared in the later stage.^[24,25]^ In this study, although the B+C recombinant subtype did not enter the network, it also accounted for 4.6%. Concern should also be paid to subtype B and recombinant subtypes. The results of this study also show that CRF01_AE, CRF07_BC and B, The three subtypes are not interconnected and cluster with each other. It indicates that the genetic distance among the three is greater than the optimal genetic distance, and the transmission risk among each other is less than that within each subtype. The CRF01_AE subtype propagation cluster ratio of CRF07_BC and B have more propagation clusters. This indicates that there are many mutations in CRF01_AE subtype, forming multiple sources and thus forming multiple transmission clusters. CRF07_BC formed only one propagation cluster and the degree of association was ≥2. Therefore, CRF07_BC subtypes are monitored dynamically over a long period of time to enable precise prevention and control measures to block their transmission.

The results showed that the factors affecting HIV-1 entry into the molecular transmission network were occupation, sample source, genotype and drug resistance. Among them, workers, testing consultation, other patient testing, subtype B and drug resistance were higher than other groups. The main sexual transmission into molecular transmission network in Liaoning Province is homosexual transmission. Risk factors for homosexual HIV infection include occupation and sample source, as reported by Shang Hong's team.^[26]^ The results showed that only 3 primary resistance mutations entered the molecular transmission network. Two of them were CRF01_AE subtype, resistance gene is protease inhibitor-related resistance, mutation site is L33F, Q58E. The L33F mutation constitutes interconnection in the largest spreading cluster in the network. Mutations K65R, Y181C+G190S, which produced high drug resistance, did not enter the transmission network.

The epidemiological characteristics of HIV-infected individuals within each molecular transmission cluster showed that 97% of the subjects were from different urban areas in the same city. The traffic between different urban areas is convenient. It has certain spatial aggregation. Studies have shown that homosexual and heterosexual transmission are positively correlated with spatial aggregation.^[27]^ The genetic distance of each molecular transmission cluster also indicates that the infected strains have high homology.

## 5 Conclusion

In short, molecular communication networks can assess the risk of transmission through the network access rate and the degree of association. And can be based on the construction of molecular network to early warning of communication risk.^[28]^ Network analysis can provide more accurate basis for prevention and control. It is suggested that the network analysis of molecular methods should be combined with social science and public health methods. Precise intervention should be carried out for high-risk groups to curb the spread of HIV.

This study has some limitations. A molecular transmission cluster only represents a group of highly associated infected individuals, and does not reflect the real transmission relationship. The retrospective analysis used in this study shows that the sample size is limited. The conclusion can only be used to observe the publicity, intervention and testing effect of antiviral treatment and voluntary counseling and testing in the past. On this basis, molecular network detection should be carried out to achieve accurate prevention and control of HIV.

## Author contributions

**Data curation:** Shan Pan.

**Formal analysis:** Ning Ma.

**Funding acquisition:** Wen-qing Yao.

**Methodology:** Ning Ma.

**Project administration:** Ning Ma.

**Resources:** Ning Ma.

**Software:** Xing-hua Chen.

**Supervision:** Ning Ma.

**Validation:** Ning Ma, Yan Zhao, Xu Kang.

**Visualization:** Ning Ma, Xing-hua Chen.

**Writing – original draft:** Ning Ma.

**Writing – review & editing:** Ning Ma.
