# HIV-1 molecular transmission network among sexually transmitted populations in Liaoning Province, China: Erratum

**DOI:** 10.1097/MD.0000000000026853

**Published:** 2021-08-06

In the article, “HIV-1 molecular transmission network among sexually transmitted populations in Liaoning Province, China”,^[1]^ which appears in Volume 100, Issue 28 of *Medicine*, Figure 1 has been replaced with the below figure with the correct figure legend.

**Figure 1:**
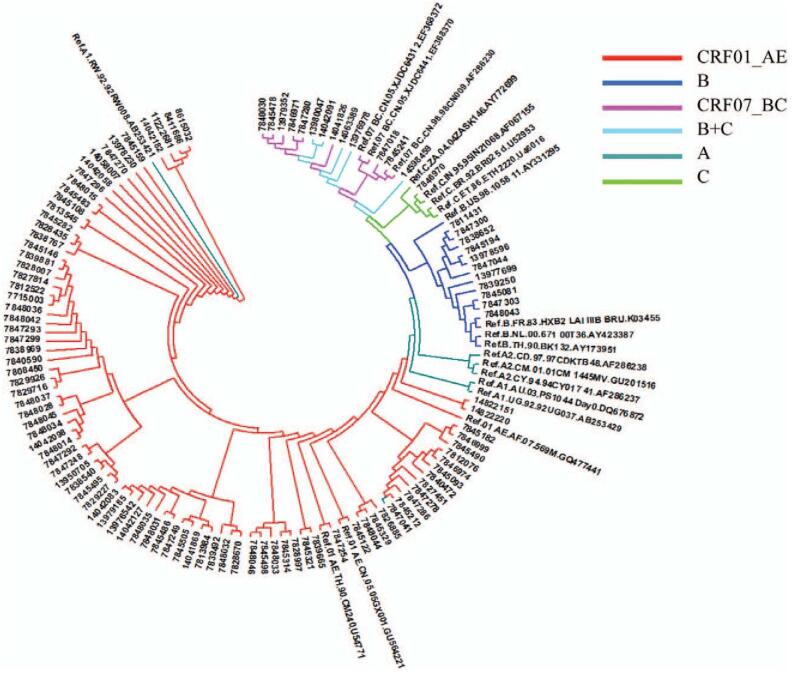
Phylogenetic tree of HIV-1 subtype adjacency method. HIV = human immunodeficiency virus.
